# Neostigmine and atropine as a treatment for postdural puncture headache after spinal anesthesia in cesarean section: A case report

**DOI:** 10.1002/ccr3.8132

**Published:** 2023-11-02

**Authors:** Indra Kumar Shrestha, Rupak Chalise, Saroj Poudel, Ashim Regmi, Anup Ghimire, Bikash Khadka, Kishor Khanal

**Affiliations:** ^1^ Critical Care Medicine Nepal Mediciti Lalitpur Nepal

**Keywords:** atropine, epidural blood patch, intensive care unit, neostigmine, PDPH, spinal anesthesia

## Abstract

**Key Clinical message:**

Neostigmine and atropine offer a promising treatment option for postdural puncture headache (PDPH) following spinal anesthesia in cesarean section, providing effective relief with a favorable risk–benefit profile.

**Abstract:**

Postdural puncture headache (PDPH) is a common consequence of cesarean section surgeries after spinal anesthesia. This case study describes the successful treatment of PDPH with intravenous neostigmine and atropine. A 31 years female who underwent elective cesarean section with spinal anesthesia developed a severe headache on the 6th postoperative day and was diagnosed to have PDPH. PDPH failed to respond to conventional treatment modalities like hydration, a Non‐steroidal anti‐inflammatory drug, and sphenopalatine ganglion block. Epidural blood patch could not be performed due to lack of consent. A trial dose of intravenous neostigmine (20 mcg/kg) along with atropine (10 mcg/kg) successfully provided symptomatic and clinical relief. The combination of neostigmine and atropine demonstrates a rapid onset of action, providing patients with effective analgesia while avoiding the need for invasive procedures such as epidural blood patches and offers quicker pain relief. This promising result warrants additional research.

## Graphical abstract

Understanding the pathophysiology of postdural puncture headache and the mechanisms of action of neostigmine and atropine for treatment.

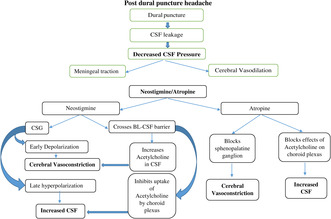

## 1 INTRODUCTION

An unpleasant experience for both the patient and the anesthetist, postdural puncture headache (PDPH) is a complication of spinal anesthesia or lumbar puncture. It is believed to be caused by cerebral vasodilation, which is an indirect consequence of low cerebrospinal fluid (CSF) pressure, or meningeal traction linked to low CSF pressure.^1^ PDPH incidence varies, although it is generally thought to be 36% or more after lumbar puncture, 0%–10% after spinal anesthesia, and 81% after an unintentional dural puncture during epidural insertion.^2^, ^3^ Although PDPH typically resolves on its own, it can make it difficult for mothers to care for their infants and lengthen hospital stays. Serious side effects such as subdural hematoma, convulsions, sagittal sinus thrombosis, and cranial nerve palsies are more infrequently linked to PDPH.

## 2 CASE PRESENTATION

We report a case of a 31‐year‐old female who had a cesarean section under spinal anesthesia with a history of gestational hypertension and presented with severe positional headache, blurring of vision on the 5th postoperative day (POD) to a local hospital where she had done her cesarean section. The headache was postural, mainly in the front‐occipital area, and worsened with upright posture. Conservative management for headache was done in primary hospital but could not subside. So, she was referred to our hospital.

On the 7th POD, she was admitted to our hospital with a worsening headache despite conservative and Non‐steroidal anti‐inflammatory drug (NSAID) treatment. During the presentation, she had difficulty speaking, and diplopia, and her Glasgow Coma Scale (GCS) was E4V5M6.

On the 8th POD, the headache was persistent with self‐reporting NRS score of 7/10 even though she was on intravenous fluids, NSAIDs, and opioids. A Sphenopalatine ganglion block was tried but that helped only for a few minutes. The patient was planned for epidural blood patch but refused to have the procedure because of her bad prior experience with spinal anesthesia.

As an alternative to EBP, on the next day, intravenous neostigmine (20 mcg/kg) along with atropine (10 mcg/kg) was given over a period of 10 min. After 30 min of injection, her pain scoring (NRS) was 1/10, and she did not require any forms of pain medication for 24 h.

## 3 DISCUSSION

A thorough history and physical examination, as well as the clinical presentation (with documented Dural puncture and acute postural headache being the most distinctive features), are used to make the diagnosis of PDPH. An intracranial pathology such as an intracranial subdural hematoma and posterior reversible encephalopathy syndrome are included in the differential diagnosis of PDPH in an obstetric patient, along with caffeine withdrawal, headaches, meningitis, sinus‐related conditions, preeclampsia, pneumocephalus, and meningitis.^4^

The International Headache Society (IHS) defines PDPH as a headache occurring within 5 days of a lumbar puncture, caused by cerebrospinal fluid (CSF) leakage through the dural puncture. It is usually accompanied by neck stiffness and/or subjective hearing symptoms within 2 weeks; the PDPH typically goes away on its own or after the leak has been sealed with an autologous epidural lumbar patch.^5^

Treatment options can be divided into conservative, pharmacological, and epidural blood patch (EBP). Conservative management has traditionally involved bed rest and fluids, though there is little evidence to support either of these measures.

Numerous other reports exist in the literature with promising results for a variety of other pharmacological agents, including 5HT agonists (e.g., Sumatriptan), gabapentin, DDAVP, theophylline, and hydrocortisone. To date, there is insufficient evidence to support their use. A recent Cochrane review has concluded that therapeutic EBP is beneficial compared with conservative treatment for PDPH.^6^ Even though it is considered the gold standard of treatment, but the success rate is 50%, and the need for a second EBP may be 40%.^7^ Early complications include backache during injection, fever, bradycardia, and seizures. Late complications include meningitis, subdural hematoma, arachnoiditis, and radicular pain.^8^ In this case, we have tried pharmacological means such as Neostigmine (20 mcg/kg) and atropine (10 mcg/kg) after conservative management failed.^9^ The numeric Rating Scale was 1/10 after 30 min of injection. She did not require any pain medications after a single dose of neostigmine and atropine. A single dose of neostigmine and atropine was enough, although this can be given every 8 h if the headache has not subsided.

**FIGURE 1:**
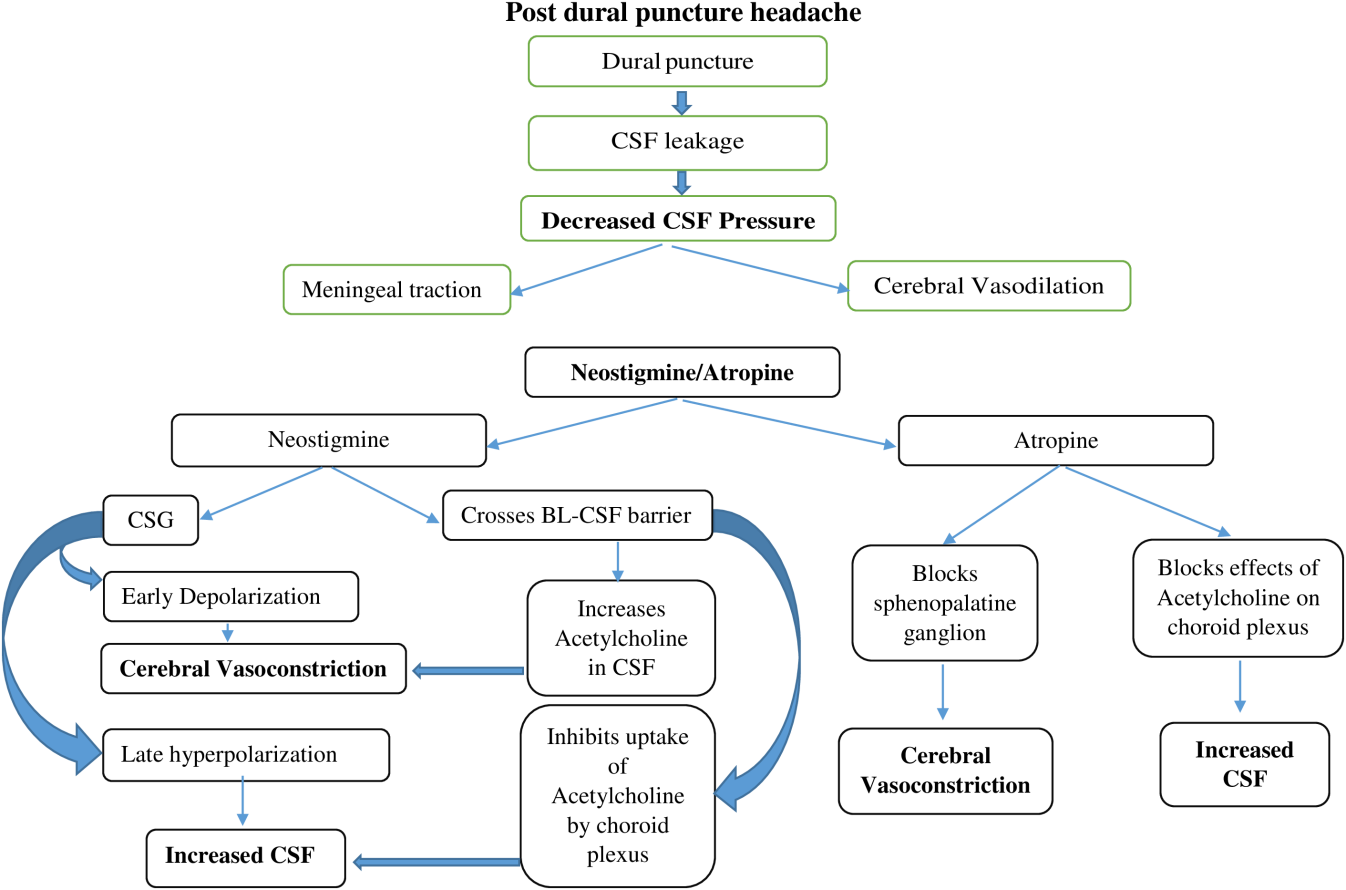
Pathophysiology of postdural puncture headache and the mechanisms of action of neostigmine and atropine treatment.

Systemic neostigmine does not cross the blood–brain barrier. However, it can enter the CSF because the BBB and Blood–CSF barriers are anatomically distinct. Neostigmine increases the level of acetylcholine in CSF and in the brain through inhibition of cholinesterase, resulting in cerebral vasoconstriction. The central effects of both drugs influence both cerebrospinal fluid secretion and cerebral vascular tone, which are the primary pathophysiological changes in PDPH (Figure 1).^10^, ^11^, ^12^, ^13^

As described by Ahmed et al in a randomized controlled trial of involving 85 patients, use of neostigmine/atropine for PDPH treatment when compared with conservative treatment of hydration and analgesic had significantly better outcome. However, side effects such as abdominal cramps, muscle twitches and urinary bladder hyperactivity occurred in the treatment group. Although the study was designed to administer the interventional drug eight hourly for a duration of 72 h, the authors report not needing more than two subsequent dosages of the treatment drug for symptomatic relief. In our case, a single dose was sufficient to achieve excellent symptomatic relief with no reported side effects.^14^

## 4 CONCLUSION

In the context of severe headache not subsiding with conservative management and intensity of headache persistently worsening, one must have a neurologic examination followed by neuroimaging for timely diagnosis and treatment. Neostigmine/atropine is a choice drug for the management of severe headaches in postdural puncture headaches after spinal anesthesia. Neostigmine/atropine was effective in treating PDPH after only a single dose. Although lacking robust evidence, use of a novel and non‐invasive treatment when compared to an invasive procedure like an epidural blood patch certainly warrants more attention and future research.

## AUTHOR CONTRIBUTIONS

**Indra Kumar Shrestha:** Conceptualization; data curation; methodology; writing – original draft; writing – review and editing. **Rupak Chalise:** Data curation; methodology; writing – original draft; writing – review and editing. **Saroj Poudel:** Data curation; methodology; writing – original draft; writing – review and editing. **Ashim Regmi:** Supervision; writing – original draft; writing – review and editing. **Anup Ghimire:** Supervision; writing – review and editing. **Bikash Khadka:** Supervision; writing – review and editing. **Kishor Khanal:** Supervision; writing – review and editing.

## FUNDING INFORMATION

None.

## CONFLICT OF INTEREST STATEMENT

No potential conflict of interest relevant to this article was reported.

## ETHICS STATEMENT

This case report did not require the approval of any Ethical Committee.

## CONSENT

Written informed consent was taken from the patient.

## Data Availability

The data used in this study are available from the corresponding author upon reasonable request.
